# Bisphenol A Exposure May Induce Hepatic Lipid Accumulation via Reprogramming the DNA Methylation Patterns of Genes Involved in Lipid Metabolism

**DOI:** 10.1038/srep31331

**Published:** 2016-08-09

**Authors:** Zhang-Hong Ke, Jie-Xue Pan, Lu-Yang Jin, Hai-Yan Xu, Tian-Tian Yu, Kamran Ullah, Tanzil Ur Rahman, Jun Ren, Yi Cheng, Xin-Yan Dong, Jian-Zhong Sheng, He-Feng Huang

**Affiliations:** 1 The Key Laboratory of Reproductive Genetics, Ministry of Education (Zhejiang University), Hangzhou, Zhejiang, China; 2 Reproductive Medicine Center, First Affiliated Hospital of Wenzhou Medical University, Wenzhou, Zhejiang, China; 3 Department of Pathology and Pathophysiology, School of Medicine, Zhejiang University, Hangzhou, Zhejiang, China; 4 International Peace Maternity and Child Health Hospital, School of Medicine, Shanghai Jiao Tong University, Shanghai, China

## Abstract

Accumulating evidence suggests a role of bisphenol A (BPA) in metabolic disorders. However, the underlying mechanism is still unclear. Using a mouse BPA exposure model, we investigated the effects of long-term BPA exposure on lipid metabolism and the underlying mechanisms. The male mice exposed to BPA (0.5 μg BPA /kg/day, a human relevant dose) for 10 months exhibited significant hepatic accumulation of triglycerides and cholesterol. The liver cells from the BPA-exposed mice showed significantly increased expression levels of the genes related to lipid synthesis. These liver cells showed decreased DNA methylation levels of *Srebf1* and *Srebf2,* and increased expression levels of *Srebf1* and *Srebf2* that may upregulate the genes related to lipid synthesis. The expression levels of DNA methyltransferases were decreased in BPA-exposed mouse liver. Hepa1-6 cell line treated with BPA showed decreased expression levels of DNA methyltransferases and increased expression levels of genes involved in lipid synthesis. DNA methyltransferase knockdown in Hepa1-6 led to hypo-methylation and increased expression levels of genes involved in lipid synthesis. Our results suggest that long-term BPA exposure could induce hepatic lipid accumulation, which may be due to the epigenetic reprogramming of the genes involved in lipid metabolism, such as the alterations of DNA methylation patterns.

The incidence of obesity and the obesity-associated metabolic disorders has dramatically risen over the last few decades, and the mechanisms remain to be illustrated. In recent years, the role of food contaminants acting as endocrine-disrupting chemicals in metabolic disorders is gaining credence. Bisphenol A (BPA), one of the highest volume chemicals produced worldwide, is an endocrine disruptor that is extremely prevalent in our environment. It is applied as the monomer of polycarbonate plastics and epoxy resins, and, is widely used in many consumer products. People are widely exposed to low levels of BPA primarily by leaching from food and beverage containers through diets, and 92.6% of urine samples collected from the National Health and Nutrition Examination Survey (NHANES III) cohort exhibited detectable BPA levels^1^. The estimates of daily average BPA intake in adults are 0.4–1.4 μg BPA/kg body weight per day^2^. It has been demonstrated that very low doses of BPA could affect endpoints of experimental studies, and different protocols usually induce inconsistent results^3^.

Mounting epidemiologic studies have revealed the association of BPA exposure and metabolic disorders, such as cardiovascular diseases, diabetes and liver damage^4^. In consistent, piles of experimental studies observed the deleterious effects of ecologically relevant doses of BPA on multiple metabolic organs including liver^5^,^6^,^7^,^8^,^9^. In rodents, both of perinatal and adult BPA exposure could lead to hepatic lipid accumulation^4^,^10^. One group administered adult male mice with doses (5, 50, 500, and 5,000 μg/kg/day) of BPA for both 4 weeks^4^ and 8 months^9^, and observed disruption of hepatic lipid metabolism in BPA-exposed mice with both protocols. However, the 4-week BPA exposure did not predict well the chronic effects, emphasizing the necessity of the model of longer BPA exposure better reflecting the ubiquitous and chronic BPA exposure in human beings.

Environmental impacts during mammalian development can cause changes in the epigenome, persisting through meiosis, which will increase the susceptibility of individual to chronic metabolic diseases^11^. Emerging clinical studies have reported the association between aberrant DNA methylation and the severity of nonalcoholic fatty liver disease (NAFLD) in patients^12^,^13^,^14^. Animal studies also linked DNA methylation to hepatic steatosis^15^,^16^. Molecular mechanisms that underlie DNA methylation-related hepatic lipid accumulation are not thoroughly illustrated, and, the role of DNA methylation in BPA-induced hepatic steatosis remains to be examined^17^.

To date, perinatal and short-term BPA exposure has been the focus of most experimental studies. For the best of human applicability, we established a mouse model of chronic human relevant BPA exposure from the first day when mice were born throughout their whole lives. We compared the metabolic indexes of 8-weeks and 10-months old male mice between control and BPA exposure groups, and, analyzed the gene expression levels of the key players in hepatic lipid metabolism to illustrate the mechanisms underlying the dyslipidemia and hepatic lipid accumulation after long-term BPA exposure. We also examined the roles of DNA methylation in the modulation of hepatic lipid metabolism. Furthermore, we examined the transcription of DNA methyltransferases to find out the possible causes of the aberrant DNA methylation patterns. To explore the mechanistic link between changes in the expression and changes in the DNA methylation, we examined whether BPA treatment in Hepa1-6 mouse hepatocyte cell line might directly alter the expression of DNA methyltransferases, and, whether knockdown of DNMTs could affect the DNA methylation and expression of genes involved in lipid synthesis. We also explored the effect of BPA treatment on the expression of genes involved in lipid synthesis in Hepa1-6, and, the effect of *Srebf1* and *Srebf2* knockdown on the transcription of genes involved in lipid synthesis.

## Results

### Long-term BPA Exposure Induced Obesity in Mice

The male mice in BPA group were exposed to 2.5 μg/L BPA from the first day they were born until the day they were sacrificed. The mice from the control and BPA groups were killed at the age of 8 weeks (young mice) and at the age of 10 months (older mice), respectively. We found that there were no significant differences in the body weight, liver weight, liver-to-body weight ratio, perigonadal white adipose tissue weight and adipose-to-body weight ratio of the young mice between the BPA group (BPA exposure for 8 weeks) and control group (Supplementary Fig. 1A–E). However, In the older mice, the body weight, liver weight, liver-to-body weight ratio, perigonadal white adipose tissue weight and the adipose-to-body weight ratio were significantly increased in the BPA group (Supplementary Fig. 1F–J), suggesting that long-term (10 months) BPA exposure might induce obesity in mice.

### Long-term BPA Exposure Altered the Homeostasis of Glucose and Lipid Metabolism in Mice

In young mice, we found that the serum total cholesterol level in the BPA group was higher than that in the control group, and there were no differences in fasting blood glucose, serum insulin and lipid status between BPA and control groups (Table 1). However, in the older mice, the BPA group showed obviously decreased HDL level and increased fasting blood glucose, serum insulin, TG, TC and LDL levels (Table 1).

### Long-term BPA Exposure Lead to Hepatic Lipid Accumulation in Mice

**Figure 1:**
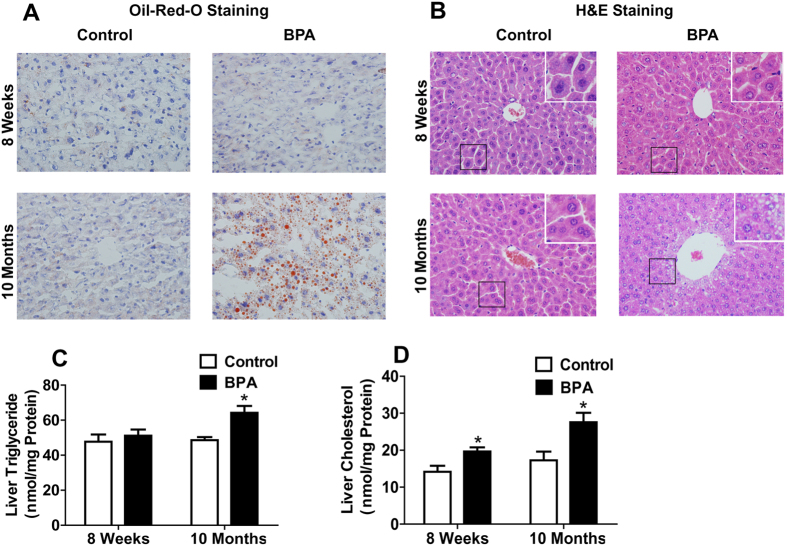
BPA-exposed Male Mice Exhibited Hepatic Lipid Accumulation in Their Middle Age. Representative photomicrographs of Oil-Red-O-stained (**A**) and H&E stained (**B**) liver sections (original magnification 400×). Hepatic content of triglyceride (**C**) and cholesterol (**D**) normalized to protein concentrations. (N, control = 7; BPA = 5 in 8-week mice. N, control = 10; BPA = 7 in 10-month mice). Values are mean ± SE. *P < 0.05 and **P < 0.01 compared with the corresponding control; Student’s *t* test.

To investigate the effect of long-term BPA exposure on hepatic lipid accumulation, we applied Oil-Red-O to stain the hepatic neutral lipids. In the young mice, there was no apparent difference in the Oil-Red-O staining between two groups. In the older mice, an increased level of Oil-Red-O staining with larger and more numerous lipid droplets in liver tissues was detected in BPA group (Fig. 1A). The data of H&E staining validated the results of Oil-Red-O staining (Fig. 1B). In young mice, the quantification of liver lipid content showed an exclusive elevation of hepatic TC content in BPA group (Fig. 1C). In older mice, both hepatic TG and TC levels were significantly increased (Fig. 1D). On the other hand, we did not detect significant differences in the serum levels of ALT and AST, that are markers of liver damage, between two groups at 8 weeks and 10 months old (Table 1).

### Long-term BPA Exposure Altered the Expression levels of the Genes Related to Lipid Metabolism in Livers of Mice

**Figure 2:**
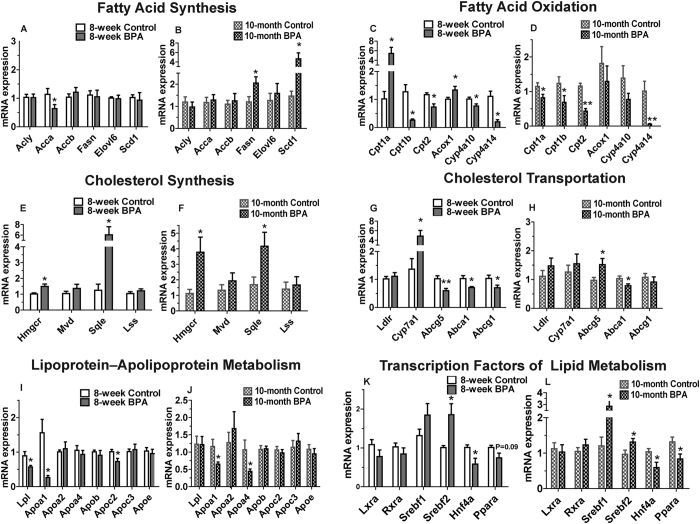
The Expression of Genes Involved in Lipid Metabolism is Altered in Livers from BPA-exposed Male Mice. Gene expression (q-PCR) of genes involved in fatty acid biosynthesis (**A,B**), fatty acid oxidation (**C,D**), cholesterol synthesis (**E,F**), cholesterol transportation (**G,H**), lipoprotein–apolipoprotein metabolism (**I,J**) and transcription factors modulating lipid metabolism (**K,L**) from 8-week (**A,C,E,G,I,K**) and 10-month (**B,D,F,H,J,L**) male mice (N, control = 7; BPA = 5 in 8-week mice. N, control = 10; BPA = 7 in 10-month mice). Values are mean ± SE. *P < 0.05 and **P < 0.01 compared with the corresponding control; Student’s *t* test.

In order to gain insight into the mechanisms underlying the effects of BPA on lipid metabolism, we examined the expression levels of genes related to fatty acid synthesis (Fig. 2A,B), fatty acid oxidation (Fig. 2C,D), cholesterol synthesis (Fig. 2E,F), cholesterol transportation (Fig. 2G,H) and lipoprotein-apolipoprotein metabolism (Fig. 2I,J). The genes related to fatty acid synthesis we examined in this study included *Acca* (Acetyl-CoA carboxylase), *Acly* (ATP citrate lyase), *Accb* (Acetyl-CoA carboxylase beta), *Fasn* (fatty acid synthase), *Scd1* (stearoylCoA desaturase 1) and *Elovl6* (elongation of very-long-chain fatty acids protein 6). We found that, compared with the control group, young mice in the BPA group showed a decreased expression level of *Acca* in liver (Fig. 2A). However, compared with the control group, older mice in the BPA group showed that the expression levels of *Fasn*, a rate-limiting enzyme of the lipogenic pathway, and *Scd1*, a central lipogenic enzyme catalyzing the synthesis of monounsaturated fatty acids, were significantly increased (Fig. 2B).

The genes related to free fatty acid oxidation we examined in this study included *Cpt1a, Cpt1b* and *Cpt2* (the subtypes of carnitine palmitoyltransferases), *Acox1* (Acyl-CoA oxidase 1), *Cyp4a10* and *Cyp4a14* (two major cytochromes P450) in the liver. We found that, compared with the control group, young mice in the BPA group showed the significantly elevated expression levels of *Cpt1a* and *Acox1* and the significantly decreased expression levels of *Cpt1b, Cpt2, Cyp4a10* and *Cyp4a14* (Fig. 2C). Compared with the control group, older mice in the BPA group showed that the expression levels of *Cpt1a, Cpt1b, Cpt2* and *Cyp4a14* were significantly reduced (Fig. 2D).

The genes related to cholesterol synthesis we examined in this study included *Hmgcr* (3-hydroxy-3-methyl glutaryl coenzyme A reductase), *Mvd* (mevalonate (diphospho) decarboxylase), *Sqle* (squalene epoxidase), and *Lss* (lanosterol synthase). We found that, compared with the control group, young and older mice in the BPA group showed the significantly increased expression levels of *Hmgcr* and *Sqle* (Fig. 2E,F).

The genes related to cholesterol transportation we examined in this study included *Ldlr* (low density lipoprotein receptor), *Cyp7a1* (cytochrome P450, family 7, subfamily A, polypeptide 1), *Abca1* (ATP-binding cassette transporter A1), *Abcg1* and *Abcg5*. We found that, compared with the control group, young mice in the BPA group showed the significantly increased expression level of *Cyp7a1* and significantly decreased expression levels of *Abcg5, Abca1* and *Abcg1* (Fig. 2G). In older mice, compared with the control group, the BPA group showed significantly increased expression level of *Abcg5* and significantly reduced expression level of *Abca1* (Fig. 2H).

The genes related to lipoprotein-apolipoprotein metabolism we tested in this study included *Lpl* (lipoprotein lipase), *Apoa1, Apoa2, Apoa4, Apob, Apoc2, Apoc3* and *Apoe* (apolipoprotein). We found that, compared with the control group, young mice in the BPA group showed the significantly decreased expression levels of *Lpl, Apoa1* and *Apoc2* (Fig. 2I). In older mice, compared with the control group, the BPA group showed significantly reduced expression levels of *Apoa1* and *Apoa4* (Fig. 2J).

### BPA Exposure Significantly Upregulated the Expression Levels of Transcription Factors *Srebf1* and *Srebf2* in Livers of Mice

Fatty acid and cholesterol metabolism in the liver is modulated by a cascade of upstream transcription factors. We investigated the expression levels of the transcription factors modulating d*e novo* fatty acid synthesis including *Lxra* (liver X receptor-alpha), *Rxra* (the retinoid X receptor-alpha) and their immediate downstream target sterol regulatory element binding protein (*Srebf1*), and the transcription factor modulating cholesterol synthesis*, Srebf2*. We found that, compared with the control group, older mice in the BPA group showed three-fold increase in the expression level of *Srebf1*, while no obvious difference was found in *Lxra* or *Rxra* expression levels (Fig. 2K,L). The expression level of *Srebf2* was significantly increased in the BPA mice of both ages (Fig. 2K,L).

We further explored the expression levels of hepatic nuclear factor 4 alpha (*Hnf4a*) and the peroxisome proliferator-activated receptor alpha (*Ppara*). Hnf4a mediates the transcription of numerous genes involved in lipid metabolism and transport, including Ppara and a bunch of apolipoproteins^18^. In younger mice, only *Hnf4a* expression level was significantly reduced in the BPA group, and, in older mice, both *Hnf4a* and *Ppara* expression levels were significantly decreased (Fig. 2K,L).

### DNA Methylation of Genes Involved in Lipid Synthesis was Downregulated by Reduced DNA Methyltransferases in Liver Samples from BPA Exposed Mice

**Figure 3:**
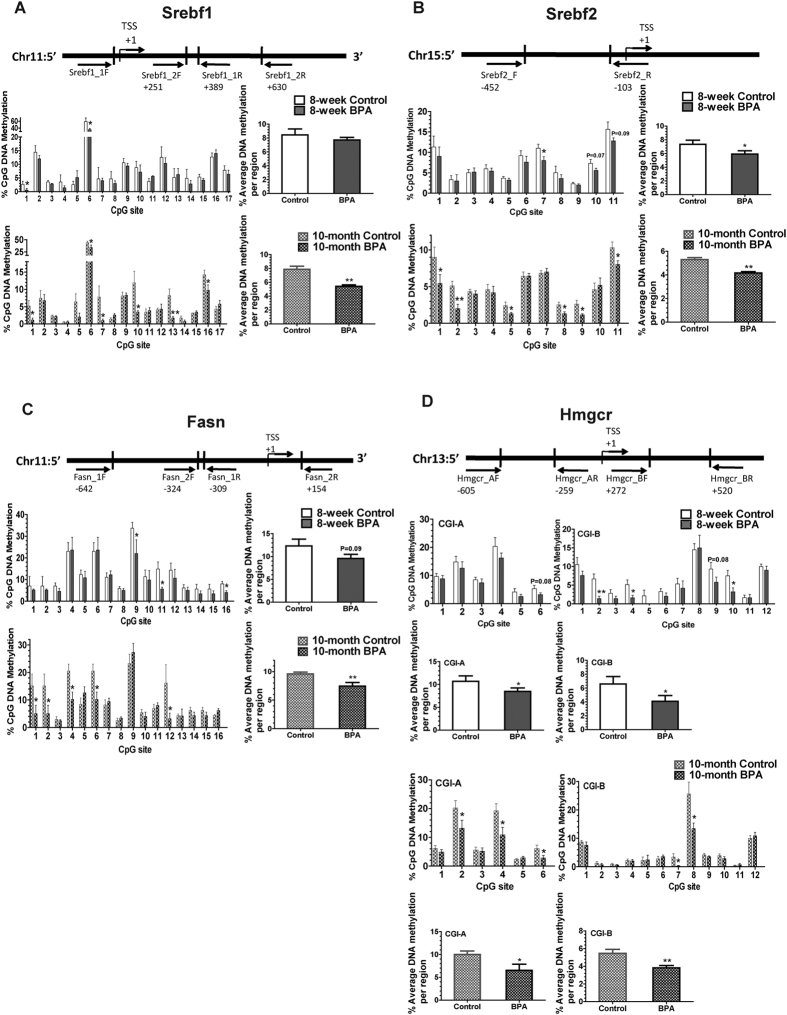
DNA Methylation of Genes Involved in Lipogenesis is Altered in Liver Samples from BPA-exposed Male Mice. The schematic diagram of amplicons in the promoter region, the mean methylation levels for individual CpG site and the average DNA methylation levels for the whole region of promoter of (**A**) *Srebf1,* (**B**) *Srebf2*, (**C**) *Fasn* and (**D**) *Hmgcr* from 8-week and 10-month male mice (N, control = 7; BPA = 5 in 8-week mice. N, control = 10; BPA = 7 in 10-month mice). *Srebf1, Srebf2* and *Fasn* have one CpG island, respectively, and *Hmgcr* has two CpG islands including CGI-A and CGI-B. Binding sties for each of the forward and reverse primers are shown as arrows below the diagram. Values are mean ± SE. *P < 0.05 and **P < 0.01 compared with the corresponding control; Student’s *t* test.

We explored the DNA methylation patterns of *Srebf1* and *Srebf2. Srebf1* and *Srebf2* both contain a canonical CpG island (CGI) around the transcription start site. The average DNA methylation levels were significantly decreased in CGI of *Srebf2* in the young BPA mice, and, in CGIs of both *Srebf1* and *Srebf2* in the older BPA mice (Fig. 3A,B). In the young BPA-exposed mice, the methylation levels of the CpG sites 1, 6 of *Srebf1 and* CpG site 7 of *Srebf2* were statistically decreased (Fig. 3A,B). In older mice, the methylation levels of the CpG sites 1, 6, 7, 10, 13, 16 of *Srebf1* and CpG sites 1, 2, 5, 8, 9, 11 of *Srebf2* were significantly reduced in the BPA group (Fig. 3A,B). It is worth noting that average DNA methylation level of *Srebf2* reduced with aging in both BPA and control groups, and, the BPA group maintained hypomethylation compared with the control in both ages.

We further explored the DNA methylation of *Fasn* and *Hmgcr*, which are the downstream targets of *Srebf1* and *Srebf2*, respectively. Fasn is the key enzyme of fatty acid and Hmgcr is the key enzyme of cholesterol synthesis. *Fasn* obtains a CGI in 5′ regulatory regions, and the average methylation was only significantly reduced in older mice. DNA methylation of CpG sites 9, 11, 16 of *Fasn* in younger mice and CpG sites 1, 2, 4, 6, 12, 16 in older mice showed a statistical reduction in the BPA group (Fig. 3C). Two CGIs were identified in the 5′ proximal region of *Hmgcr*, and both showed significant reduction of average DNA methylation levels in the BPA-exposed mice of both ages. CpG sites 2, 4, 6 of CGI-A in older mice and CpG sites 2, 4, 10 of CGI-B in young mice and CpG sites 7, 8 of CGI-B in older mice showed significantly decreased DNA methylation levels in the BPA group (Fig. 3D). In agreement with *Srebf2*, the average DNA methylation levels of *Fasn* and *Hmgcr* also decreased with aging in both groups.

**Figure 4:**
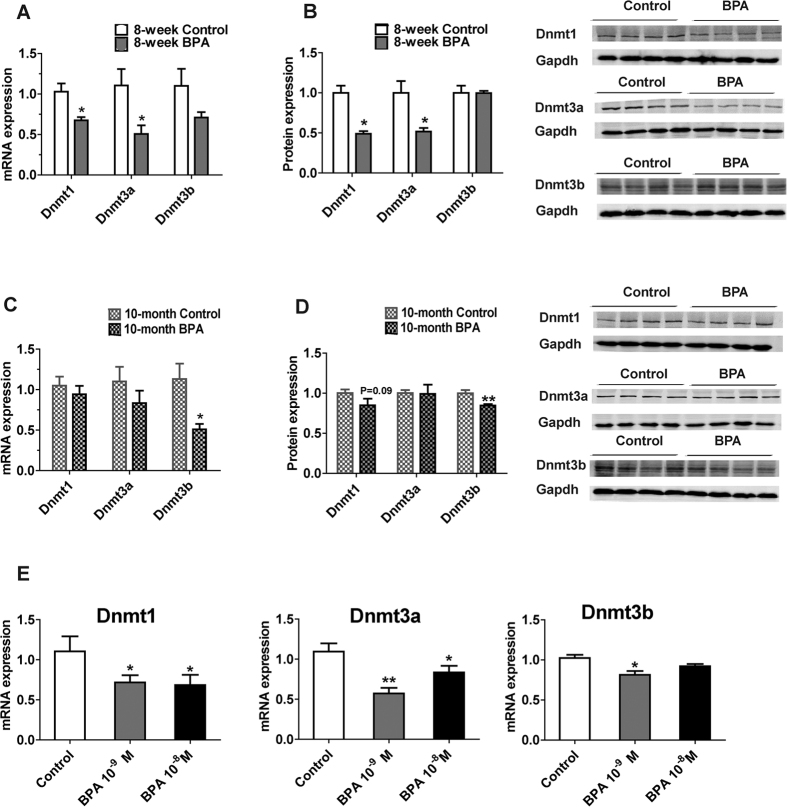
The Expression of DNA Methyltransferases is Altered in Livers from BPA-exposed Male Mice and BPA-treated Hepa1-6 cell line. Gene expression (qPCR) of DNA methyltransferases from (**A**) 8-week mice and (**C**) 10-month mice (N, control = 7; BPA = 5 in 8-week mice. N, control = 10; BPA = 7 in 10-month mice.). Protein expression (western blotting) of DNA methyltransferases from (**B**) 8-week mice and (**D**) 10-month mice (N = 4). Gene expression (q-PCR) of *Dnmt1, Dnmt3a* and *Dnmt3b* (**E**) in Hepa1-6 mouse hepatocyte cell line (N = 4) treated with BPA (10^−9^M and 10^−8^M). Values are mean ± SE. *P < 0.05 and **P < 0.01 compared with the corresponding control; Student’s *t* test.

DNA methylation patterns are established in early mammalian development and maintained throughout the lifetime of the organisms. Dnmt1, that prefers hemi-methylated DNA substrate, is responsible for maintenance of DNA methylation after DNA replication. Dnmt3a and Dnmt3b, responsible for *de novo* methylation, are also actively involved in the maintenance of DNA methylation^19^. We examined the expression levels of DNA methyltransferases (*Dnmt1, Dnmt3a* and *Dnm3b*) to explore the mechanism underlying the hypo-methylation of lipogenesis genes. In young mice, both mRNA and protein expression levels of Dnmt1 and Dnmt3a were significantly reduced (Fig. 4A,B). In older mice, the BPA group showed a reduced level of Dnmt3b (Fig. 4C,D).

We treated Hepa1-6 mouse hepatocyte cell line with BPA. BPA at 10^−9^M downregulated the expression of *Dnmt1, Dnmt3a* and *Dnmt3b,* and BPA at 10^−8^M downregulated the expression of *Dnmt1* and *Dnmt3a* (Fig. 4E).

### DNA Methyltransferase Knockdown Led to Hypo-methylation of Genes Involved in Lipid Synthesis in Hepa1-6 Mouse Hepatocyte Cell Line

**Figure 5:**
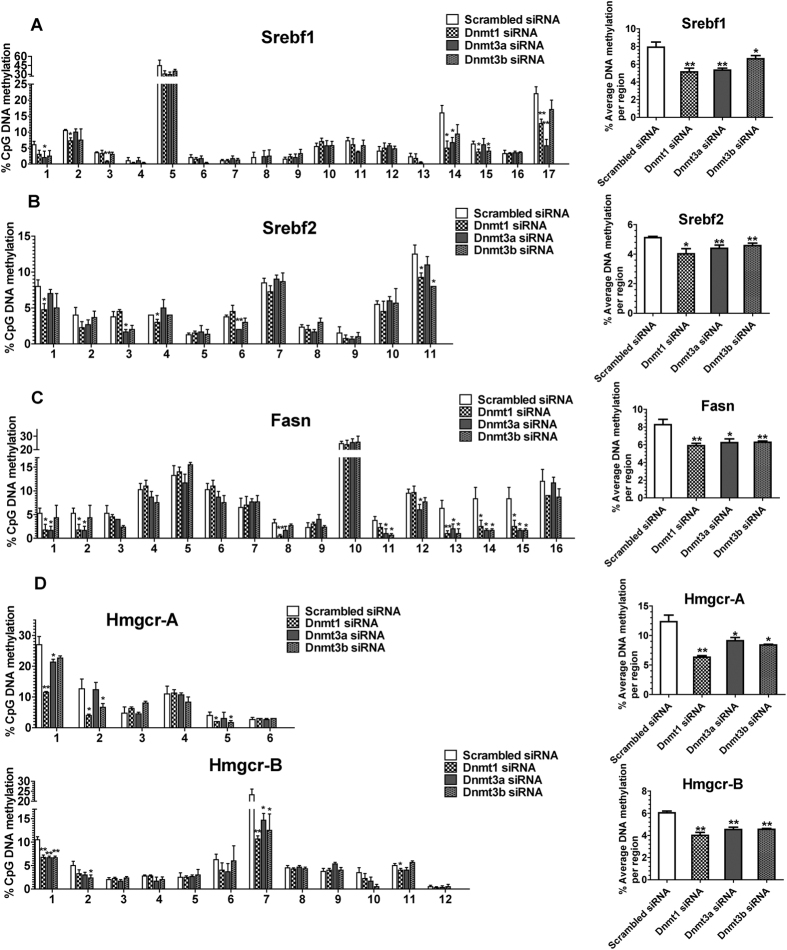
Effects of DNA Methyltransferase Knockdown on the DNA Methylation Levels of Genes Involved in Lipid Synthesis in Hepa1-6 Cells. The average methylation levels for individual CpG site and the average DNA methylation levels for the whole region of promoter of (**A**) *Srebf1,* (**B**) *Srebf2*, (**C**) *Fasn* and (**D**) *Hmgcr* in in Hepa1-6 mouse hepatocyte cell line (N, control/ Dnmt1siRNA = 4; Dnmt 3a/3b siRNA = 3). *Srebf1, Srebf2* and *Fasn* have one CpG island, respectively, and *Hmgcr* has two CpG islands including CGI-A and CGI-B. Values are mean ± SE. *P < 0.05 and **P < 0.01 compared with the corresponding control; Student’s *t* test.

To further explore the mechanisms underlying the changes in DNA methylation of lipogenesis gene promoters, we treated Hepa 1-6 (a mouse hepatocyte cell line) with the DNMT siRNAs. All three siRNAs targeted to *Dnmt1, Dnmt3a* and *Dnmt3b*, respectively, led to substantial downregulation of DNMT expression two days after the initial transfection (Supplementary Fig. 2A–C). Knockdown of *Dnmt1, Dnmt3a* and *Dnmt3b* induced significant reduction of the average methylation levels of *Srebf1, Srebf2, Fasn* and *Hmgcr* (Fig. 5A–D). In all four genes involved in lipid synthesis, *Dnmt1* knockdown showed the greatest reduction of overall promoter methylation followed by *Dnmt3a* knockdown, and, *Dnmt3b* knockdown showed the smallest reduction. Knockdown of *Dnmt1* significantly decreased the DNA methylation levels of CpG sites 1, 3, 14, 15, 17 of *Srebf1*, CpG sites 1, 4, 11 of *Srebf2*, CpG sites 1, 2, 8, 13, 14, 15 of *Fasn*, CpG sites 1, 2, 5 of CGI-A of *Hmgcr* and CpG sites 1, 7, 11 of CGI-B of *Hmgcr*. Knockdown of *Dnmt3a* significantly reduced the DNA methylation levels of CpG sites 1, 3, 14, 17 of *Srebf1*, CpG sites 3, 6 of *Srebf2*, CpG sites 1, 2, 11, 12, 13, 14, 15 of *Fasn*, CpG sites 1of CGI-A of *Hmgcr* and CpG sites 1, 7 of CGI-B of *Hmgcr*. Knockdown of *Dnmt3b* significantly decreased the DNA methylation levels of CpG sites 15 of *Srebf1*, CpG sites 11 of *Srebf2*, CpG sites11, 13, 14, 15 of *Fasn*, CpG sites 2, 5 of CGI-A of *Hmgcr* and CpG sites 1, 2, 7 of CGI-B of *Hmgcr* (Fig. 5A–D).

**Figure 6:**
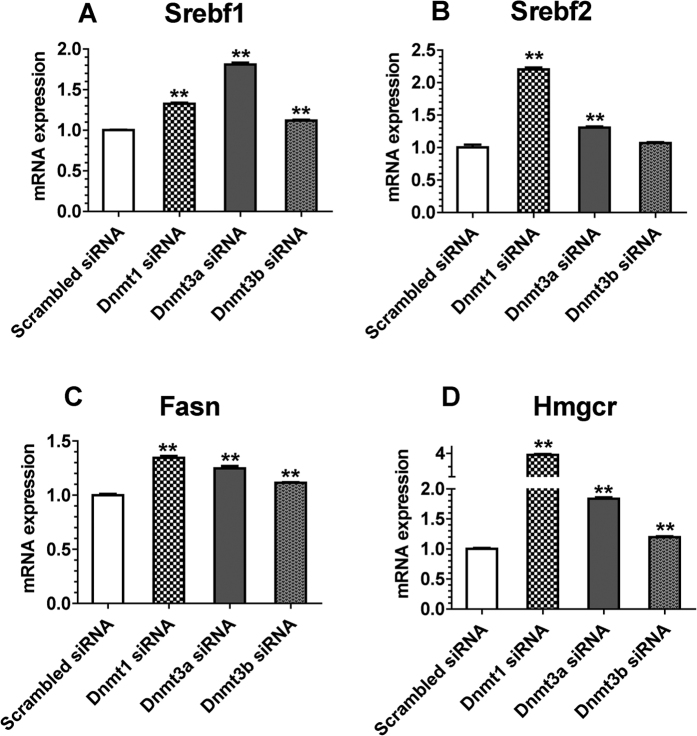
Effects of DNA Methyltransferase Knockdown on the mRNA Expression of Genes Involved in Lipid Synthesis in Hepa1-6 Cells. Gene expression (qPCR) of *Srebf1* (**A**), *Srebf2* (**B**), *Fasn* (**C**) and *Hmgcr* (**D**) in Hepa1-6 mouse hepatocyte cell line (N = 4). Values are mean ± SE. *P < 0.05 and **P < 0.01 compared with the corresponding control; Student’s *t* test.

We also investigated the effect of DNA demethylation on mRNA expression of lipogenesis genes. *Dnmt1* knockdown and *Dnmt3a* knockdown both significantly upregulated the expression of all four lipogenesis genes, while *Dnmt3b* knockdown upregulated the mRNA levels of *Srebf1, Fasn* and *Hmgcr* (Fig. 6A–D). Furthermore, *Dnmt1* knockdown obviously increased the levels of triglyceride and cholesterol in Hepa1-6 cells and *Dnmt3a* knockdown increased the level of triglyceride only (Supplementary Fig. 3A,B).

### BPA Treatment Upregulated the Expression of Genes Involved in Lipid Synthesis in Hepa1-6 Cells

**Figure 7:**
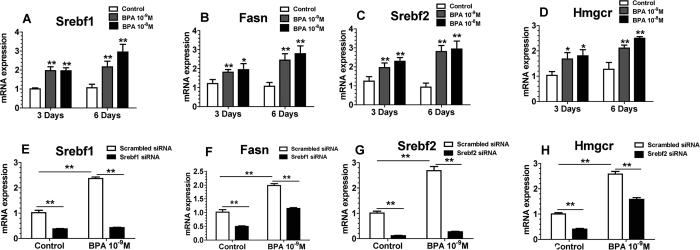
The Effect of BPA Treatment and Srebf1 and Srebf2 Knockdown on the Expression of Genes Involved in Lipid Synthesis in Hepa1-6 Cells. Gene expression (qPCR) of *Srebf1* (**A**), *Fasn* (**B**), *Srebf2* (**C**) and *Hmgcr* (**D**) in Hepa1-6 mouse hepatocyte cell line (N = 4) treated with BPA (10^−9^M and 10^−8^M). Gene expression (qPCR) of *Srebf1* (**E**), *Fasn* (**F**), *Srebf2* (**G**) and *Hmgcr* (**H**) in Hepa1-6 mouse hepatocyte cell line (N = 4) treated with both BPA (10^−9^M) and siRNA. Values are mean ± SE. *P < 0.05 and **P < 0.01 compared with the corresponding control; Student’s *t* test.

In order to investigate the association between BPA action and the relations between Srebf1 and Srebf2 and their downstream targets, we treated Hepa1-6 mouse hepatocyte cell line with BPA, Srebf1 siRNA and Srebf2 siRNA. BPA at 10^−9^M and 10^−8^M both upregulated the expression of *Srebf1, Fasn, Srebf2* and *Hmgcr* (Fig. 7A–D). The increasing fold of all four genes in Hepa1-6 treated with BPA for 6 days was greater than that in Hepa1-6 treated with BPA for 3 days.

Both siRNAs targeted to *Srebf1* and *Srebf2* respectively led to substantial downregulation of *Srebf1* and *Srebf2* expression in Hepa1-6 cell line treated with DMSO (vehicle) or 10^−9^M BPA (Fig. 7E–H). In Hepa1-6 cells treated with DMSO or BPA, knockdown of *Srebf1* lead to decreased expression of *Fasn*, and, knockdown of *Srebf2* decreased *Hmgcr* expression (Fig. 7E–H). The downregulation of *Fasn* expression by *Srebf1* knockdown and downregulation of *Hmgcr* expression by Srebf2 knockdown were compromised by BPA treatment compared with the control (treated with DMSO).

## Discussion

Recently, piling evidence validated the role of BPA in metabolism. Yet short-term and perinatal exposure is often the focus of most experimental studies, and the results are inconsistent due to different protocols. For the best of human applicability, we conducted this mouse model to evaluate the effects of long-term, oral BPA exposure on metabolic disorders. The mice in this study were administered with either 0 or 2.5 μg BPA/L drinking water. Our preliminary study confirmed a mean body weight of 20 g and a daily intake of about 4 milliliter water in adult mice, that was earlier established by another group^2^. Therefore, the BPA dose in this study was estimated as daily consumption of 0.5 μg BPA/kg BW, that was similar to the BPA concentration the human daily exposed to.

The middle-aged male mice in the present study exhibited obesity, glucose intolerance, dyslipidemia, and, hepatic accumulation of triglycerides and cholesterol after long-term (10 months) BPA exposure. We examined the expression profile of the genes related to hepatic lipid metabolism. It showed that the mechanisms potentially underlying the aberrant hepatic lipid metabolism and associated dyslipidemia were increased fatty acid and cholesterol synthesis, impaired triglyceride and cholesterol transportation, and reduced fatty acid oxidation. In the older mice, the expression levels of key enzymes of fatty acid and cholesterol synthesis were significantly increased in the BPA exposure mice. The mean level of *Fasn* in the BPA exposure mice was almost 2 times of that in the control mice, and, the levels of *Scd1, Hmgcr* and *Sqle* in the BPA group were 4 times of those in the control group. These data suggest that the activation of fatty acid and cholesterol synthesis may be important mechanisms underlying the aberrant lipid metabolism. The major role of aberrant lipid synthesis in BPA-induced disorders is in agreement with earlier studies^4^,^9^. However, in the present study we selected the lower BPA dose and longer BPA exposure time compared with other studies. In the two-hit theory of nonalcoholic fatty liver disease (NAFLD) to explain the pathogenesis of nonalcoholic steatohepatitis (NASH), the triglyceride accumulation constitutes the first “hit” in NASH that is needed for the development of this disease. Specially, excessive *de novo* fatty acid synthesis is now believed to facilitate the production of lipotoxic lipid intermediates that contribute to the pathogenesis of NASH^20^. On the other hand, increased cholesterol levels are recognized as a risk factor for ischemic heart disease and coronary mortality. Collectively, the hepatic lipid accumulation induced by BPA exposure may lead to more severe pathologies such as NASH and cardiac events, so it should be carefully examined.

In our model, the effects of BPA on the expression of genes involved in lipid metabolism differ between young and old mice in many cases. *Cpt1a* and *Acox1*, the key players of fatty acid oxidation, were significantly upregulated in the younger BPA mice and downregulated in the older BPA mice. The expression level of *Cyp7a1*, the key player of cholesterol export from hepatocytes to bile, was increased in the younger BPA mice and showed no difference between BPA and control groups in older mice. The exclusive upregulation of *Cpt1a, Acox1* and *Cyp7a1* in the younger BPA mice might be due to some ‘feedback regulation’ mechanisms existed only in the younger BPA mice against excessive triglyceride and cholesterol. Meanwhile, the expression changes of many genes (including *Srebf1, Fasn, Scd1, Hmgcr, Cpt2, Cyp4a14, Hnf4a* and *Ppara*) induced by BPA exposure were more significant in the older BPA mice than younger animals. Altogether, the aberrant expression of these genes may lead to metabolic disorders in older mice.

Srebf1 and Srebf2 activate the complete program of fatty acid and cholesterol synthesis in the liver^21^. In Hepa1-6 cell line treated with DMSO (vehicle) or 10^−9^M BPA, knockdown of *Srebf1* or *Srebf2* lead to decreased expression of *Fasn* and *Hmgcr*, respectively. Also, Srebf1 and Srebf2 both downregulate the hepatic expression of *Hnf4a*^22^. Hnf4a mediates the transcription of numerous genes involved in lipid metabolism and transport, including Ppara and a bunch of apolipoproteins^18^. BPA-exposed mice in this study showed increased transcription of *Srebf1* associated with increased *Fasn,* and, *Srebf2* with *Hmgcr*. We also found that *Hnf4a* was decreased together with *Ppara* and several apolipoproteins (*Apoa1, Apoa4* and *ApoC2*). Meanwhile, the expression levels of downstream molecules of *Ppara* including *Lpl, Abca1, Abcg1* and fatty acid oxidation pathway (*Cpt1, Acox1* and *Cyp4a*) were also decreased. Taken together, the increased expression levels of *Srebf1* and *Srebf2* induced by BPA may play the critical role in the hepatic lipid accumulation.

*Srebf1* is mainly modulated at the transcriptional level by Lxra controlled by insulin^23^. The expression of *Lxra* remained unchanged regardless of increased insulin in the BPA mice of middle age. This might be due to the insulin resistance in liver, that is strongly linked to NAFLD^20^. The expression of *Srebf2* is primarily modulated by the ‘convergent feedback inhibition’ of cellular sterol level^21^. However, it kept the statistical increase of transcription despite hepatic cholesterol accumulation in the BPA group. The increasing fold of expression level of *Srebf1* in the BPA group is different from its downstream target *Fasn*. This difference also existed in *Srebf2* and its downstream target *Hmgcr*. In Hepa1-6 cell line, the downregulation of *Fasn* expression by *Srebf1* knockdown and downregulation of *Hmgcr* expression by *Srebf2* knockdown were compromised by BPA treatment. These findings strongly suggested the involvement of other mechanisms in the excessive transcription of SREBPs and their targeted genes. In general consensus, promoter methylation levels tend to relate negatively to gene expression. Promoters of *Srebf1, Srebf2, Fasn* and *Hmgcr* were hypomethylated in the BPA-exposed mice, which is very likely to contribute to the promoted transcription of *Srebf1* and *Srebf2* and their targets. As mentioned before, the transcriptional changes of *Srebf1* and many SREBPs-regulated genes induced by BPA exposure progressed with aging. In Hepa1-6 cell line, the transcriptional changes of *Srebf1, Srebf2, Fasn* and *Hmgcr* induced by BPA treatment also progressed with treatment duration. These results suggested that BPA- induced DNA hypo-methylation might accumulates over time and accelerates the deregulated expression patterns of genes.

A progressive loss of overall methylation has been found in aging animals^24^. In agreement, the average methylation levels of *Srebf2, Fasn* and *Hmgcr* decreased with aging in our BPA and control mice. Average DNA methylation levels of *Srebf2, Fasn* and *Hmgcr* in the BPA-exposed mice were significantly lower than the control mice. It is worth noting that average methylation levels of the three genes in younger BPA-exposed mice were comparable to the ones in older control mice. Although highly speculative, this comparability might contribute to the early-onset metabolic disorders in contemporary culture.

Emerging studies have reported the association between early-life BPA exposure and disrupted DNA methylation patterns that lead to all kinds of health problems^25^,^26^,^27^. Yet, little is known concerning the mechanism underlying. DNA methylation patterns are established in early mammalian development and maintained throughout the lifetime of the organisms. Dnmt1, that prefers hemi-methylated DNA substrate, is responsible for maintenance of DNA methylation after DNA replication. Dnmt3a and Dnmt3b, responsible for *de novo* methylation, are also actively involved in the maintenance of DNA methylation^19^. *Dnmt1* and *Dnmt3a* were significantly reduced in the young BPA-exposed mice, and *Dnmt3b* was significantly decreased in older mice. In Hepa 1-6, BPA treatment downregulated the expression of DNMTs. The DNMT knockdown lead to hypo-methylation and increased expression of the lipogenesis genes and increased lipid content in Hepa1-6. These results suggest that aberrant DNMT expression may contribute to the altered DNA methylation patterns and expression of lipogenesis genes. In all four lipogenesis genes from Hepa 1-6, *Dnmt1* knockdown showed the greatest reduction of DNA methylation followed by *Dnmt3a* knockdown, and, *Dnmt3b* knockdown showed the smallest reduction. The mRNA and protein expression levels of Dnmt1 and Dnmt3a were all almost 50% reduced in the BPA mice compared with control mice in younger animals. Taken together, our results suggest that DNA methylation may be more vulnerable to BPA exposure in early adulthood than middle age.

In conclusion, long-term human relevant dose of BPA exposure influences the expression of genes involved in hepatic lipid metabolism, thereby leading to metabolic disorder in middle-aged male mice. Reprogrammed DNA methylation patterns of genes involved in hepatic lipid synthesis, caused by disrupted expression of DNMTs, are quite likely one of the underlying mechanisms. Regarding the global effect of DNMTs, DNA methylation patterns of whole hepatic genome are quite likely to be generally modulated. The long-lasting epigenetic vulnerability in liver induced by BPA will predispose individuals to multiple health problems. In this consideration, the window of intense avoidance of BPA exposure should be elongated from perinatal period at least into early adulthood, since complete avoidance is almost impossible in the context of the ubiquitous nature of BPA^28^.To thoroughly examine the mechanisms that underlie metabolic disorders induced by BPA exposure, future studies will be needed to illustrate the mechanisms and timing of epigenetic disruption and identify the multiple signaling pathways and genes that may be involved. Additionally, future studies should include a range of human relevant doses and both sexes to properly assess the impacts of BPA on male and female metabolism.

## Methods

### Animal Care and Experimental Design

Eight-week-old female ICR (CD-1) mice were purchased from Shanghai Institutes for Biological Science (Shanghai, China). One single virgin female mouse was mated with one non-sibling male. Once pregnancy was confirmed by vaginal plug, the male mouse was removed from the cage, and the pregnant mice were kept individually throughout gestation. From the first day of childbirth and throughout lactation, mother mice were randomly assigned to two groups: the mice in the first group drank water containing 2.5 μg/L BPA (Sigma-Aldrich, St. Louis, MO, USA; estimated 0.5 μg BPA /kg/day, a human relevant dose)^2^, and, the mice in the second group drank water containing vehicle (0.1% ethanol by volume) only. On the other hand, after delivery, litters were adjusted to eight pups per dam to avoid metabolic drifts according to nutrient availability during lactation. Offspring were nursed freely and weaned at 3 weeks onto standard diet. After weaning, the male offspring from the mothers in the first group were daily administered 2.5 μg/L BPA in water similar to the BPA water that their mothers drank (BPA group) and the male offspring from the mothers in the second group were administered water with vehicle (0.1% ethanol by volume) only (control group). Protocols were approved by the Animal Ethics Committee of Zhejiang University. The methods of animal experiments in this study were carried out in accordance with the approved guidelines.

### Blood and Organ Sampling

Part of male offspring at the age of 8 weeks from each group were randomly selected, and, then weighted and sacrificed after fasting overnight. The rest mice were killed at the age of 10 months. After testing fasting blood glucose level with blood obtained from the tail vein using an Accu-check compact glucometer (Roche Diagnostic GmbH, Mannheim, Baden-Württemberg, Germany), we collected the blood from mouse eyes. Plasma was prepared by centrifugation (2000 rpm, 10 minutes) and kept at −20 °C until use. Livers and the perigonadal white adipose tissue (pWAT) were removed, weighed, dissected, snap-frozen in liquid nitrogen, and kept at −80 °C until use.

### Biochemical Assays

Hepatic content of triglyceride (TG) and total cholesterol were measured using TG and total cholesterol assay kit (Applygen Technologies Co. Ltd, Beijing, China), respectively. Lipid contents were then normalized to protein concentrations tested with protein quantitative assay kit (Thermo-Fisher, Waltham, MA, USA). Serum levels of TG, total cholesterol, low- and high-density lipoprotein (LDL, HDL) cholesterol, glutamicpyruvic transaminase (ALT) and glutamic oxalacetic transaminase (AST) were assayed using a biochemical analyzer (TBA120FR, Toshiba, Tokyo, Japan). Serum insulin was determined with the mouse insulin enzyme-linked immunosorbent assay (ELISA) kit (Crystal Chem, Downers Grove, IL, USA).

### Hepa1-6 Cell Culture and Transfection of DNMT siRNAs

The mouse hepatocyte cell line (Hepa1-6) were purchased from the cell bank of the Chinese Academy of Sciences (Shanghai, China) and maintained under standard culture conditions (DMEM, 10% fetal bovine serum). siRNAs targeting *Dnmt1, Dnmt3a* and *Dnmt3b* were purchased from Shanghai Gene Pharma Co, Ltd (Shanghai, China) and prepared as described previously^29^. The siRNA sequences against *Dnmt1* were 5-GCUGGUCUAUCAGAUCUUUTT-3 (sense) and 5-AAAGAUCUGAUAGACCAGCTT-3 (antisense); siRNA sequences against Dnmt3a were 5-GCACAACAGAGAAACCUAATT-3 (sense) and 5-UUAGGUUUCUCUGUUGUGCTT-3 (antisense); siRNA sequences against Dnmt3b were 5-CCAAGCGCCUCAAGACAAATT-3 (sense) and 5-UUUGUCUUGAGGCGCUUGGTT-3 (antisense). The sense and antisense oligonucleotides were stored at −20 °C before use. The day before transfection, Hepa 1–6 was seeded such that cells were 30–50% confluent the next day. Cells were transfected with 35 nM siRNA as protocol indicated using Lipofectamine 3000 (Life Technologies, Foster City, CA, USA) in Opti-MEM I reduced serum medium (Life Technologies) at 37 °C in a 5% CO_2_ atmosphere for 6 h. The medium was then removed and replaced with standard culture conditions. Control cells were treated with scrambled siRNA. Transfection was repeated at 2, 4, and 6 days for a total of 4 treatments as described previously^30^. Cells were harvested at 9 days after the initial transfection for additional analysis.

### BPA administration in Hepa1-6 Cell Line and Transfection of Srebf1 and Srebf2 siRNAs in Hepa1-6 Cell Line

BPA was dissolved in DMSO as concentration of 10^−5^M and 10^−6^M and then added to the culture medium (0.1% by volume) for Hepa1-6 with the final concentration of 10^−8^M and 10^−9^M. The control group was treated with culture medium containing vehicle (0.1% DMSO by volume) only. Cells cultured for 2 days were harvested for DNMT mRNA analysis and cultured for 3 and 6 days were harvested for *Srebf1, Srebf2, Fasn* and *Hmgcr* mRNA analysis.

For siRNA transfection, cells first cultured with DMSO or 10^−9^M BPA for 4 days and then transfected with Srebf1 and Srebf2 siRNAs and were harvest on the 6th day for *Srebf1, Srebf2, Fasn* and *Hmgcr* mRNA analysis. siRNAs were also purchased from Shanghai Gene Pharma Co, Ltd (Shanghai, China). The siRNA sequences against Srebf1 were 5-GGUCUUCUAUCAAUGACAATT-3 (sense) and 5-UUGUCAUUGAUAGAAGACCTT-3 (antisense); siRNA sequences against Srebf2 were 5-CAACCUCAGAUCAUUAAGATT-3 (sense) and 5-UCUUAAUGAUCUGAGGUUGTT-3 (antisense).

### Histology

For detection of lipid accumulation, frozen liver samples were embedded in Neg 50 (Fisher Scientific, Courtaboeuf, Essonne, France), sliced to 5 μm sections and stained with Oil-Red-O and hematoxylin. To observe the liver morphology, liver specimens in 4% of paraformaldehyde were embedded in paraffin, sliced to 5 μm sections and stained with H&E (hematoxylin and eosin) following routine procedures.

### DNA and RNA Extraction

Genomic DNA from tissues was extracted using TIANamp Genomic DNA Kit (Tiangen Biotech Co., Ltd, Beijing, China). Total RNA was isolated with TRIzol reagent (TaKaRa, Dalian, China).

### RT-qPCR Analysis

cDNA was synthesized from 1 μg of total RNA by reverse transcription, using RT reagent Kit (TaKaRa). The amplification of the genes that were listed in Supplementary Table S1 was performed using real-time quantitative PCR on a 7900 Real-Time PCR System (Applied Biosystems, Foster City, CA, USA) using SYBR-Green pre-mix Ex Taq (TaKaRa). The expression levels of genes were normalized to GAPDH. The list of primers was also shown in Supplementary Table S1.

### CT conversion and MassArray EpiTYPER Quantitative DNA Methylation Analysis

Genomic DNA (1000 ng) from each sample was modified by bisulfite conversion with the EZ DNA CT Conversion Reagent, Zymo Research Corporation (Irvine, CA, USA) following the manufacturer’s protocol. DNA methylation was quantified by Mass Array EpiTyper (Sequenom, San Diego, CA, USA) as described previously (Ehrich *et al*.). The promoter regions of each target gene were analyzed. Amplicons cover the regions of the relevant CpG sites and the target regions are shown in the relevant figures. Primers were designed by the online software Epidesigner (http://www.epidesigner.com) and shown in Supplemental Table S2. The quantitative methylation data for one single CpG site or aggregates of multiple sites obtained from Mass Array were analyzed using the EpiTYPER software (Sequenom).

### Western Blot Analysis

Western blots were performed using total protein extract as previously described^30^. Gapdh protein levels were used as control for equal protein loading. The list of antibodies is available in Table S3.

### Statistical Analysis

Results are presented as mean ± SE (n is the number of tissue preparations). We applied the software SPSS version 19.0 software (SPSS Inc., Chicago, IL, USA) to perform statistical analysis. The comparisons between two groups were performed with Student’s *t* test. A *P*-value < 0.05 was considered statistically significant.

## Additional Information

**How to cite this article**: Ke, Z.-H. *et al*. Bisphenol A Exposure May Induce Hepatic Lipid Accumulation via Reprogramming the DNA Methylation Patterns of Genes Involved in Lipid Metabolism. *Sci. Rep.* **6**, 31331; doi: 10.1038/srep31331 (2016).

## Supplementary Material

Supplementary Information

## Figures and Tables

**Table 1:** Basal Serum Glucose, Insulin, TG, TC, HDL, LDL, ALT and AST in Male Mice.

	8-week Male Mice		10-month Male Mice	
	Control (n = 7)	BPA (n = 5)	Control (n = 10)	BPA (n = 7)
Glucose (mmol/L)	5.08 ± 0.42	4.97 ± 0.32	5.47 ± 0.36	6.90 ± 0.80**
Insulin (μg/L)	0.91 ± 0.07	0.96 ± 0.08	0.85 ± 0.22	1.17 ± 0.10**
TG (mmol/L)	0.92 ± 0.24	0.86 ± 0.35	1.47 ± 0.42	2.16 ± 0.42**
TC (mmol/L)	1.92 ± 0.23	2.31 ± 0.19*	2.55 ± 0.25	2.88 ± 0.31*
HDL (mmol/L)	1.50 ± 0.15	1.87 ± 0.36	1.67 ± 0.16	1.47 ± 0.19*
LDL (mmol/L)	0.69 ± 0.08	0.61 ± 0.21	0.60 ± 0.12	0.77 ± 0.19*
ALT (U/L)	30.42 ± 7.08	22.60 ± 6.65	33.40 ± 12.40	42.71 ± 16.64
AST (U/L)	256.83 ± 19.81	250.20 ± 27.44	182.00 ± 48.43	209.57 ± 81.03

TG, triacylglycerol; TC, total cholesterol; HDL, high density lipoprotein; LDL, low density lipoprotein; ALT, glutamic-pyruvic transaminase; AST, glutamic oxalacetic transaminase. Values are mean ± SE. *P < 0.05 and **P < 0.01 compared with the corresponding control; Student’s *t* test.
