# Supplementary material for: Bisphenol A Exposure May Induce Hepatic Lipid Accumulation via Reprogramming the DNA Methylation Patterns of Genes Involved in Lipid Metabolism
Source: Sci Rep. 2016 Aug 9;6:31331. doi: 10.1038/srep31331 (PMC4977563; doi:10.1038/srep31331)
Supplement: Supplementary Information [file srep31331-s1.pdf]

Title Page

Bisphenol A Exposure May Induce Hepatic Lipid Accumulation via Reprogramming the DNA Methylation Patterns of Genes Involved in Lipid Metabolism

Zhang-Hong Ke[1, *], Jie-Xue Pan [1,4, *], Lu-Yang Jin[1,2], Hai-Yan Xu[1], Tian-Tian Yu[1], Kamran Ullah[1,2], Tanzil Ur Rahman[1,2], Jun Ren[1,2], Yi Cheng [1,2], Xin-Yan Dong[1,2], He-Feng Huang[1,3, #], Jian-Zhong Sheng[1,2, #]

[1]The Key Laboratory of Reproductive Genetics, Ministry of Education (Zhejiang University), Hangzhou, Zhejiang, China; [2]Department of Pathology and Pathophysiology, School of Medicine, Zhejiang University, Hangzhou, Zhejiang, China; [3]International Peace Maternity and Child Health Hospital, School of Medicine, Shanghai Jiao Tong University, Shanghai, China and [4]Reproductive Medicine Center, First Affiliated Hospital of Wenzhou Medical University, Wenzhou, Zhejiang, China

* Both authors contributed equally to the study.

Corresponding Author: Jian-Zhong Sheng, Ph.D., Department of Pathology and Pathophysiology, School of Medicine, Zhejiang University, Hangzhou 310058, China. Tel: +86-571-88208803. Fax: +86-571-88208022. E-mail: shengjz@zju.edu.cn; or He-Feng Huang, MD, The International Peace Maternity and Child Health Hospital, School of Medicine, Shanghai Jiao Tong University, Shanghai 200030, China. Tel: +86-21-64073897. Fax: +86-21-64078219. E-mail: huanghefg@hotmail.com

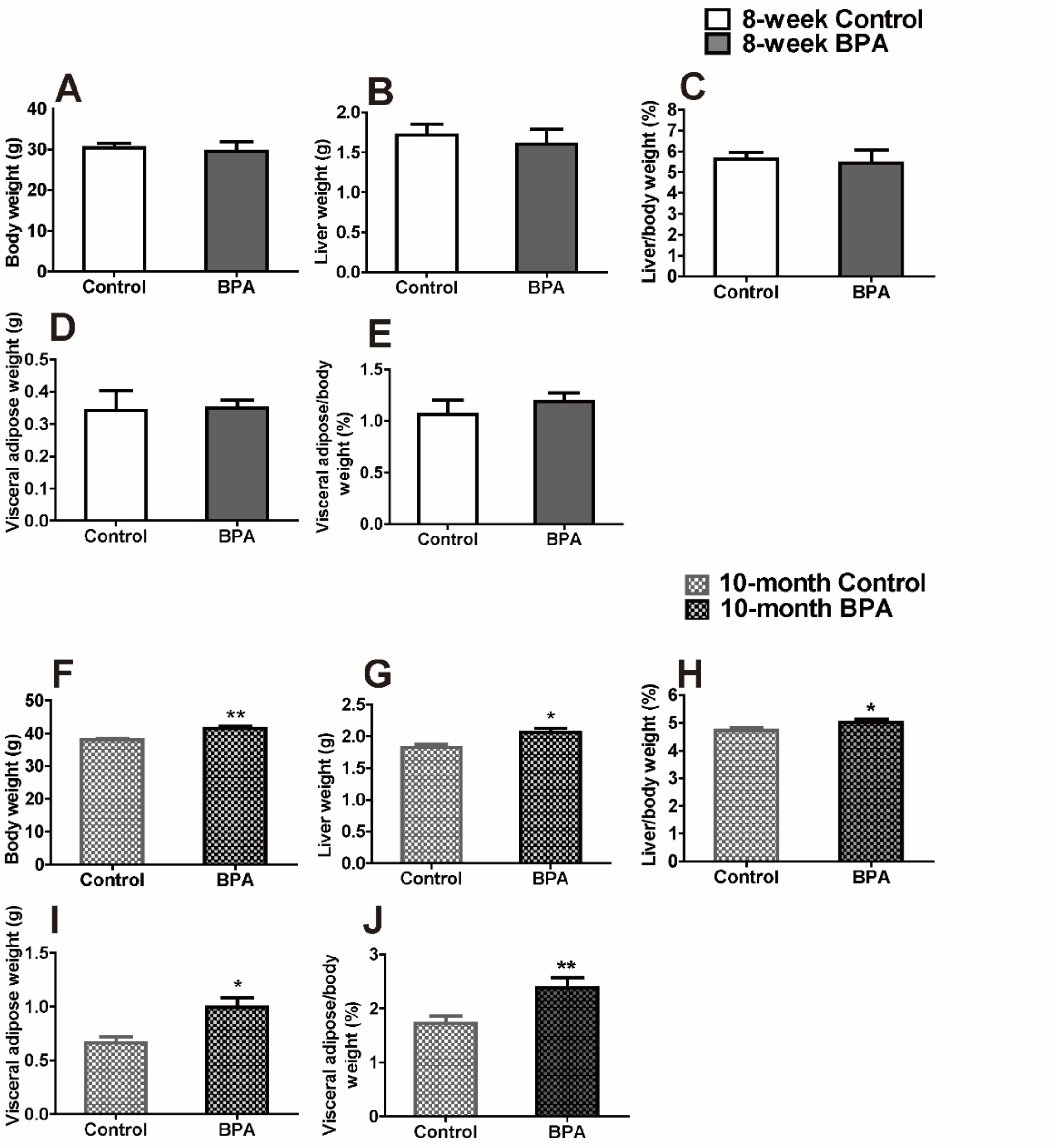

Supplemental Figure 1. The Effect of Chronic BPA Exposure on Body, Liver and Visceral Adipose Weight in Male Mice. Body weight, liver weight, liver-to-body weight ratio, perigonadic white adipose tissue weight and adipose-to-body weight ratio from 8-week (A-E) and 10-month (F-J) male mice (N, control=7; BPA=5 in 8-week mice. N, control=10; BPA=7 in 10-month mice.).Values are mean ± SE. *, $P < 0.05$ and **, $P < 0.01$ compared with the corresponding control; Student's *t* test.

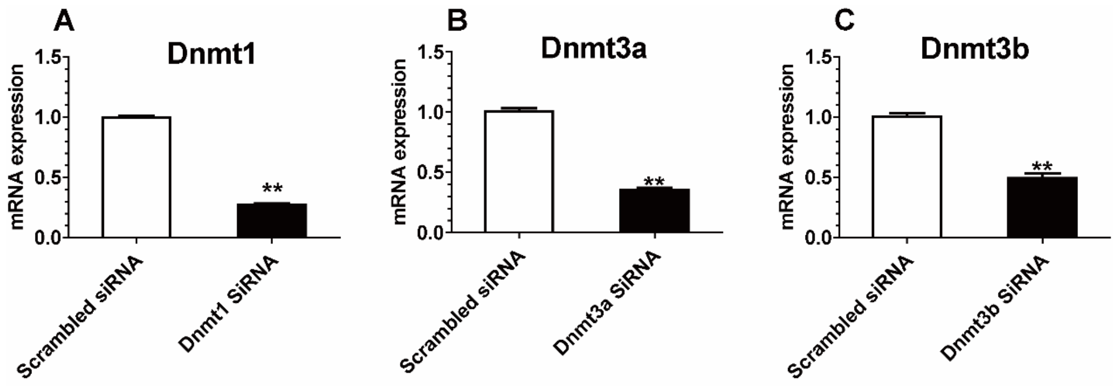

Supplemental Figure 2. mRNA Expression of DNA methyltransferases in Hepa1-6 Cells Transfected with Scrambled-siRNA and Specific siRNA Targeting DNA Methyltransferases, Respectively. Gene expression (q-PCR) of *Dnmt1*(A), *Dnmt3a* (B) and *Dnmt3b* (C) in Hepa1-6 mouse hepatocyte cell line (N=4). Values are mean ± SE. *, $P < 0.05$ and **, $P < 0.01$ compared with the corresponding control; Student's *t* test.

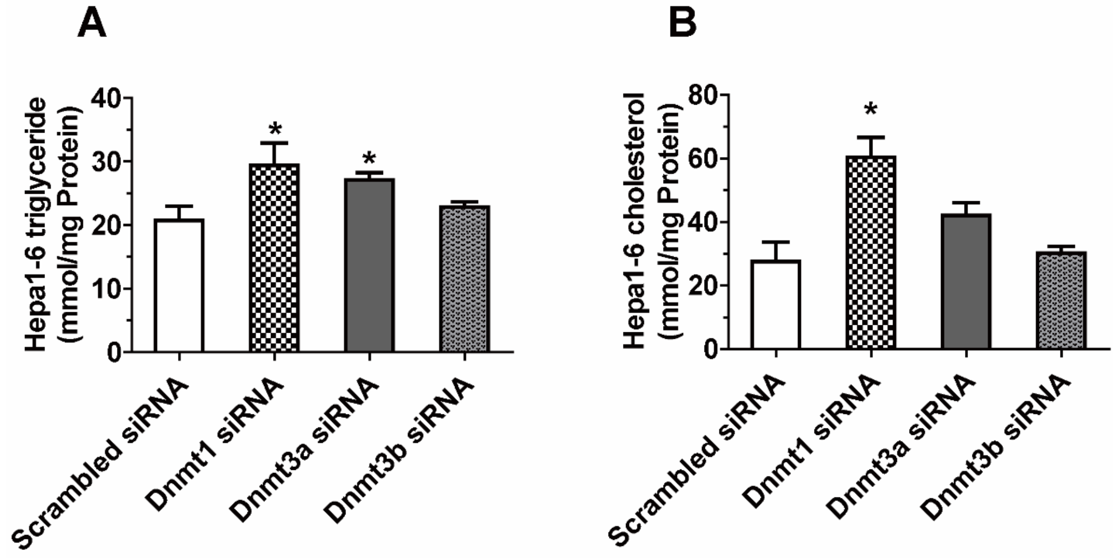

Supplemental Figure 3. Effects of DNA Methyltransferase Knockdown on Triglyceride and Cholesterol Content in Hepa1-6 Cells. Content of triglyceride (A) and cholesterol (B), normalized to protein concentrations, in Hepa1-6 mouse hepatocyte cell line (N, Scrambled/Dnmt1 siRNA=4, Dnmt3a/3b siRNA=3). Values are mean ± SE. *, $P < 0.05$ and **, $P < 0.01$ compared with the corresponding control; Student's *t* test.

Supplemental Table 1. Primers for Real-time PCR

Lipid biosynthesis	
Acca_F	TTACAGGATGGTTTGGCCTTTC
Acca_R	CAAATTCTGCTGGAGAAGCCAC
Accb_F	CCAGTCTTCCGTGCCTTTGTAC
Accb_R	CTCATCCCTCGCTCTGAACG
Acly_F	CAGCCAAGGCAATTTCAGAGC
Acly_R	CTCGACGTTTGATTAACTGGTCT
Elovl6_F	TCTGATGAACAAGCGAGCCA
Elovl6_R	TGGTCATCAGAATGTACAGCATGT
Fasn_F	GGAGGTGGTGATAGCCGGTAT
Fasn_R	TGGGTAATCCATAGAGCCCAG
Scd1_F	CCGGAGACCCCTTAGATCGA
Scd1_R	TAGCCTGTAAAAGATTTCTGCAAACC
Cholesterol biosynthesis	
Mvd_F	CGGTCAACATCGCAGTTATCAA
Mvd_R	GTGCAGCGTGACGCTCAG
Hmgcr_F	CTTGTGGAATGCCTTGTGATTG
Hmgcr_R	GAAGAATGTCATGAACACAAAGTAGTTG
Lss_F	ATGAGTTGGGTCGGCAGAGAT
Lss_R	GCGCTTTTGGTAAGTCCGTG
Sqle_F	GGAGGCTACCGTGTTCTCCA
Sqle_R	CTGCACTTGGTTGGTTTCTGAC
Fatty acid oxidation	
Cpt1a_F	TCTACACCTTGAGGATGGTTCC
Cpt1a_R	GTGGTCTTTGCTTTCCCACT
Cpt1b_F	CCAGATGGAGAGGATGTTCAACA
Cpt1b_R	AGAAGCGACCTTTGTGGTAGACA
Cpt2_F	CAGTGCACAGAAGCCTCTCTTG
Cpt2_R	CTTCCCAATGCCGTTCTCAA
Acox1_F	TCGAAGCCAGCGTTACGAG
Acox1_R	ATCTCCGTCTGGGCGTAGG
Cyp4a10_F	GCCAAATCCAGAGGTGTTTGA
Cyp4a10_R	AGCAAATTGTTTCCCAATGCA
Cyp4a14_F	TCAGTCTATTTCTGGTGCTGTTC
Cyp4a14_R	GAGCTCCTTGTCCTTCAGATGGT
Fatty acid uptake and trafficking	
CD36_F	GTTAAACAAAGAGGTCCTTACACATACAG

CD36_R	CAGTGAAGGCTCAAAGATGGC
Fabp_F	GGCAAGTACCAATTGCAGAGC
Fabp_R	AGGTCCTCGGGCAGACCTA
Fatp2_F	GGAACCACAGGTCTTCCAAA
Fatp2_R	TAAAGTAGCCCCAACCACGA
Fatp5_F	GGAACTCTACGGCTCCACAG
Fatp5_R	GGCTCTGCCGTCTCTATGTC
VLDL synthesis and secretion	
Lpl_F	CCAGAAAAGTGAATCTTGACTTGGT
Lpl_R	AAGGTCAGAGCCAAGAGAAGCA
Apoa1_F	ctggccgtggctctggtcttc
Apoa1_R	gctgtctttgaccgcatccaca
Apoa2_F	AATGGTCGCACTGCTGGTAA
Apoa2_R	TTGGCCTTCTCCACCAAATC
Apoa4_F	TTCCTGAAGGCTGCGGTGCTG
Apoa4_R	CTGCTGAGTGACATCCGTCTTCTG
Apoa5_F	AGGAACTGAGCCATCCACAC
Apoa5_R	GAGAGTCACGCCAGACCAAC
Apob_F	CTGAACATCAAGAGGGGCATC
Apob_R	GGTAACCTGAGTTGAGCAGTTT
ApoCII_F	GCAGGGCTCCCTCTTAAGTT
ApoCII_R	AAAATGCCTGCGTAAGTGCT
ApoCIII_F	GGCTGGATGGACAATCACTT
ApoCIII_R	TGGTTGGTCCTCAGGGTTAG
Apod_F	TCACCACAGCCAAAGGACAAAA
Apod_R	TTGGTTCATGGTTCCATCAGGA
Apoe_F	GCAGAGCTCCCAAGTCACACAA
Apoe_R	AGTCGGTTGCGTAGATCCTCCA
Cholesterol uptake and trafficking	
Cyp7a1_F	GCTGAGAGCTTGAAGCACAAGA
Cyp7a1_R	TTGAGATGCCCAGAGGATCAC
Lplr_F	TGTGAAAATGACTCAGACGAACAA
Lplr_R	GGAGATGCACTTGCCATCCT
Abca1_F	CGTTTCCGGGAAGTGTCCTA
Abca1_R	CTAGAGATGACAAGGAGGATGGA
Abcg1_F	TCACCCAGTTCTGCATCCTCTT
Abcg1_R	GCAGATGTGTCAGGACCGAGT
Abcg5_F	GCATCGCTGTGTATCGCAAC

Abcg5_R	CCTGCAGAGCGACGTTTTTC
Transcriptional regulations	
Hnf4a_F	CCGGGTGTCAGGAACAGTTG
Hnf4a_R	TGCAGGACAGTCTGAGCCATC
Chrebp_F	ACTCAGGGAATACACGCCTACAG
Chrebp_R	GAAGAAGGAATTCAGAGCTCAGAAA
Lxra_F	GGAGTGTCGACTTCGCAAATG
Lxra_R	TCAAGCGGATCTGTTCTTCTGAC
Rxra_F	GGGAGGGCTGGGTACTATCT
Rxra_R	AGCACCAGCTTCCAGTCAGT
Ppara_F	CCCTGTTTGTGGCTGCTATAATTT
Ppara_R	GGGAAGAGGAAGGTGTCATCTG
Pparg_F	CACAATGCCATCAGGTTTGG
Pparg_R	GCTGGTCGATATCACTGGAGATC
Pxr_F	AGAGATCATCCCTCTTCTGCCAC
Pxr_R	GATCTGGTCCTCAATAGGCAGGT
Srebf1_F	CAGCTCAGAGCCGTGGTGA
Srebf1_R	TTGATAGAAGACCGGTAGCGC
Srebf2_F	GCGTTCTGGAGACCATGGA
Srebf2_R	ACAAAGTTGCTCTGAAAACAAATCA
Insig1_F	GAGGTGTCACAGTGGGAAACATAG
Insig1_R	TCTTCATCACACCCAGGACCA
Insig2_F	TGTATATTTTTTGCTGGAGGCATAAC
Insig2_R	TTCAGCAATAACTTTGCATTCATACAT
DNA methyltransferase	
Dnmt1_F	CCTAGTTCCGTGGCTACGAGGAGAA
Dnmt1_R	TCTCTCTCCTCTGCAGCCGACTCA
Dnmt3a_F	GCCGAATTGTGTCTTGGTGGATGACA
Dnmt3a_R	CCTGGTGGAATGCACTGCAGAAGGA
Dnmt3b_F	GCGTCAGTACCCCATCAGTT
Dnmt3b_R	CACGAGGTCACCTATTCCAAA

Supplemental Table 2.

Primers for MassArray EpiTYPER Quantitative DNA Methylation Analysis

Fasn_1F	aggaagagagTTTTTAAGGTGGTTATAGAGGGTGG
Fasn_1R	cagtaatacgactcactatagggagaaggctTACACTAAAAAAAACACCAAAACCA
Fasn_2F	aggaagagagTATTTGTTTGGGTTTTGGAGGTAG
Fasn_2R	cagtaatacgactcactatagggagaaggctTATCCTCCTCAATACAAAATTTCCA
Srebf1_1F	aggaagagagAGTTGTTAGGATGTAGGTTGGTGG
Srebf1_1R	cagtaatacgactcactatagggagaaggctAATAAACTCAAAAAATAAAAAACCCC
Srebf1_2F	aggaagagagGTTTGAGATTTAGGGTTGAGTTTTT
Srebf1_2R	cagtaatacgactcactatagggagaaggctAAAAAATATTCTAAAAAACACCTTCTCTC
Hmgcr_1F	aggaagagagGGTGGGTTTGAGGGTTAGTGTAG
Hmgcr_1R	cagtaatacgactcactatagggagaaggctAACCCACTAAAAAAAACACAACCTA
Hmgcr_2F	aggaagagagTAGGTTGTGTTTTTTTTAGTGGGTT
Hmgcr_2R	cagtaatacgactcactatagggagaaggctCCAATATAAATCCAAAACTTTTACCC
Srebf2_F	aggaagagagATTTTATTTAGAGTTAGGGGGAGGG
Srebf2_R	cagtaatacgactcactatagggagaaggctTCAATAAAACCAAACTTACATTAACCA

Supplemental Table 3. Primary Antibody Information

**Antibody**	**Reference**	**Working dilution**
Dnmt1	ab13537; Abcam	1:1000
Dnmt3a	D220559; Sangon Biotech	1:2000
Dnmt3b	sc-52922; Santa Cruz	1:200
Gapdh	ab181602; Abcam	1:5000
